# Indirect participant recruitment and representativeness in healthcare workforce research: lessons from a comparative study of the SCOHPICA cohort

**DOI:** 10.1136/bmjopen-2025-110324

**Published:** 2026-06-28

**Authors:** Antoine Hennard, Leonard Roth, Chiara Storari, Emilie Zuercher, Jonathan Jubin, Annie Oulevey Bachmann, Ingrid Gilles, Isabelle Peytremann-Bridevaux

**Affiliations:** 1 Department of Epidemiology and Health Systems, Centre for Primary Care and Public Health (Unisanté), University of Lausanne, Lausanne, Switzerland; 2 La Source School of Nursing, University of Applied Sciences and Arts Western Switzerland (HES-SO), Lausanne, Switzerland; 3 Human Resources Direction, Lausanne University Hospital (CHUV), Lausanne, Switzerland

**Keywords:** Health Workforce, Health Services, REGISTRIES, Surveys and Questionnaires, Research Design

## Abstract

**Abstract:**

**Objectives:**

To assess the representativeness of participants recruited through indirect methods into a cohort of healthcare professionals in the absence of comprehensive and up-to-date national registers.

**Design:**

Comparisons of socio-demographic and socio-professional characteristics from multiple national data sources with those of a large indirectly recruited healthcare workforce cohort.

**Setting:**

National healthcare workforce research conducted in Switzerland as a case study, with lessons applicable to other high-income countries lacking comprehensive registers.

**Participants:**

Healthcare professionals across 10 professional groups, with data drawn from 14 administrative, institutional, professional and research sources collected between 2016 and 2023, alongside participants from the 2022–2023 baseline recruitment waves of the Swiss Cohort of Healthcare Professionals and Informal Caregivers (SCOHPICA).

**Main outcome measures:**

Comparisons of age, sex, spoken language, training country, activity rate, working hours and sector of practice across available datasets.

**Results:**

Broad similarities were observed in sex distribution between indirectly recruited participants and external data sources. However, discrepancies emerged in age profiles, linguistic distribution and certain profession-specific variables. These variations reflected both genuine diversity within the population and inconsistencies between available datasets.

**Conclusions:**

SCOHPICA illustrates that in the absence of comprehensive and up-to-date registers, indirect participant recruitment can provide rich, diverse and generally comparable data on healthcare professionals, though representativeness cannot be definitively established. Such investigations offer valuable insights for monitoring and planning healthcare workforces internationally while underscoring the need for improved national registers and standardised workforce indicators.

**STRENGTHS AND LIMITATIONS OF THIS STUDY**

- Triangulation of 14 administrative, institutional, professional and research sources alongside a large cohort of healthcare professionals enabled broad comparisons of socio-demographic and socio-professional characteristics across multiple healthcare professions.
- Systematic assessment of representativeness in an indirectly recruited nationwide cohort provides methodological insights for healthcare workforce research in settings lacking comprehensive and up-to-date national registers.
- Statistical testing was complemented by assessment of absolute differences in proportions, allowing interpretation to consider both statistical significance and the magnitude of observed discrepancies.
- Heterogeneity of comparative datasets limited direct comparability of socio-demographic and socio-professional variables across professions and sources.
- Absence of gold-standard registers constrained the ability to definitively assess representativeness.

## Background

 In health research, ensuring the representativeness of participants is central for obtaining valid and generalisable estimates.^1 2^ However, the quest for representativeness is not always straightforward or feasible, particularly when studying specific subgroups of the population, such as healthcare professionals. Traditional methods of recruiting participants, such as probability sampling, rely on comprehensive, up-to-date registers of the target population.^3^ While such registers are usually available for general population surveys, they may be non-existent, incomplete or contain erroneous information such as outdated postal addresses or missing email information for more specific groups like healthcare professionals. In addition, healthcare professionals are distributed across multiple professions and care settings and may be either salaried or independent, with recruitment further complicated by time constraints and administrative or organisational barriers within healthcare institutions, which have been shown to reduce participation in surveys of healthcare professionals.^4^

These issues present a serious challenge when attempting to recruit a random sample that reflects the entire target population. In this case, indirect recruitment methods, such as outreach through professional associations’ newsletters or publications, may provide an appropriate alternative.^5^ Indirect recruitment or non-probability sampling is understood here as a method of selecting participants from the target population through channels other than traditional registers. This type of recruitment does not select individuals at random; rather, it aims to gather the maximum number of participants to obtain the largest possible amount of data.

Non-probability sampling, while not without limitations, can provide a legitimate and effective alternative when other selection methods are not feasible.^5 6^ Thus, as demonstrated by the Physical Activity through Sustainable Transport Approaches (PASTA) project, indirect recruitment methods are effective in maximising the number of engaged participants.^6^ However, they tend to introduce selection biases in favour of specific sub-populations. The tension between recruitment accessibility and participant representativeness is therefore central to the issue. Kim^7^ explored the growing use of non-probability sampling methods in survey research, especially in contexts where traditional probability sampling is impractical due to cost, access or low response rates.^7^ The author concluded that non-random sampling, although it lacks the randomness required for elementary statistical inference, can nonetheless yield valuable insights. In fact, richness of data, capturing in-depth information on relevant variables, can often mitigate concerns about representativeness, particularly when the goal is not to make sweeping population-level estimates, as long as it provides a broad understanding of the targeted problem. While concerns about sample bias are valid, it should be reminded that even when random sampling methods are used, non-participants most of the time differ from participants in ways that can introduce bias.^8 9^

In the absence of comprehensive databases that could be used for random sampling, indirect recruitment methods may be the only feasible and practical option for obtaining data from healthcare professionals. In Switzerland, for instance, registers of healthcare workers remain incomplete, which complicates efforts to draw representative samples.^10^,^12^ Similar limitations are found elsewhere, as even where registers do exist, they are often fragmented and not directly comparable across regions, reinforcing the recurring reliance on non-probability recruitment.^13^

This article examines the representativeness of indirectly recruited participants in a nationwide cohort such as the Swiss Cohort of Healthcare Professionals and Informal Caregivers (SCOHPICA)^14 15^ by comparing socio-demographic and socio-professional characteristics of its 2022–2023 baseline participants with those from other Swiss data sources. In doing so, it aims to assess whether indirect recruitment can constitute a practical and scientifically informative approach for studying healthcare professionals in the absence of comprehensive and up-to-date national registers while evaluating the extent to which the resulting sample reflects the target population.

## Methods

### Study design and setting

This article presents an exploratory study on the representativeness of healthcare professionals from the SCOHPICA cohort. SCOHPICA is a nationwide longitudinal project launched in 2022, designed to track the professional trajectories, working conditions and well-being of healthcare professionals and informal caregivers over time. Participants were recruited primarily through indirect channels (eg, professional associations, institutional partners, employer networks, conferences, newsletters and social media campaigns), since no comprehensive and up-to-date national registers exist. The baseline sample analysed here includes participants from the 2022–2023 recruitment waves who are healthcare professionals (SCOHPICA-HCP). In the context of SCOHPICA and this study, healthcare professionals are defined as professionals actively engaged in clinical practice with patients. To compare data for this population, we conducted a search of existing national information sources related to healthcare professionals to collect available socio-demographic and socio-professional variables. Subsequently, we performed descriptive and basic inferential statistics to compare these variables with those included in SCOHPICA-HCP, whose data are accessible on request through a data repository.^15^

### Data search

To contextualise and assess the representativeness of SCOHPICA-HCP, we conducted an extensive multi-source search for existing Swiss studies and datasets characterising healthcare professionals, guided by methodological recommendations for scoping reviews that advocate drawing on diverse data sources to maximise coverage and reduce bias.^16^ The search strategy combined (1) publicly available statistics from national institutions (eg, the Swiss Federal Statistical Office), (2) professional association datasets (eg, Swiss Medical Association’s (FMH) physician statistics), (3) institutional reports from the Swiss Health Observatory (Obsan) and (4) nationwide academic or applied research projects (eg, the Swiss Nursing Homes Resources Project (SHURP)). Large-scale population surveys, such as the national census and the Swiss Labour Force Survey, were not retained either because of insufficiently detailed professional information or because of small sample sizes for specific healthcare professional groups.^17 18^

In addition to systematically reviewing official databases and websites, we contacted relevant professional organisations to identify unpublished or internal data. This multi-source triangulation approach, incorporating institutional reports and statistics, professional association data, academic research and grey literature, aligns with recommended best practices for comprehensive data identification in settings with fragmented data sources.^19^ It also represents a reproducible framework applicable to other countries with similarly decentralised data infrastructures.

### Inclusion criteria

Comparative sources were included if they (1) concerned healthcare professionals practising in Switzerland (eg, physicians, nurses and allied health workers), (2) reported at least one socio-demographic (eg, age and sex) and/or socio-professional variable (eg, activity rate and sector of practice) and (3) had been published between 2016 and 2023. The lower bound of 2016 was chosen to ensure inclusion of key national reports while maintaining temporal proximity to the SCOHPICA-HCP data collection period. Although the overall distribution of the healthcare workforce is not expected to change substantially annually, restricting sources to this timeframe helped ensure that comparative datasets reflected broadly similar workforce structures and definitions. Only professions represented by at least 50 SCOHPICA-HCP participants were included in comparative analyses. Research projects such as the work-related stress among health professionals in Switzerland study (STRAIN), which reported characteristics for aggregated groups of professions rather than individual professions, were excluded.^20^

### Data extraction and analysis

For each source, we extracted available socio-demographic and socio-professional data: sex, age, activity rate, number of hours worked per week, sector of practice, specialty, country of training and spoken language (the three main national languages in Switzerland being German, French and Italian). Corresponding data from SCOHPICA-HCP participants were extracted in harmonised formats (eg, aligning age categories across datasets). Each comparative source was additionally rated (i) on a five-point scale of its expected target-population representativeness, taking into account population coverage, recruitment method and participation level (5 = exhaustive administrative data; 4 = broad register or professional association statistics; 3 = large voluntary survey; 2 = moderate ample participation concerns; 1 = small or selective sample), and (ii) on a five-point similarity-of-scope scale assessing how closely the target population of the external source matched that of SCOHPICA-HCP (5 = national, all settings; 4 = national, covering multiple settings but not all; 3 = national, restricted to specific settings; 2 = subnational or institution-based population; 1 = highly specific subgroup).

Descriptive statistics were produced for each extracted variable within each professional group. Furthermore, proportions for each category of the variables were compared between external data sources and the SCOHPICA-HCP sample using two-proportion z-tests (two-sided), with statistical significance set at the 95% confidence level. To complement statistical testing and account for the influence of sample size on p-values, absolute differences in proportions were also examined. Differences exceeding 10 percentage points were considered indicative of potentially meaningful discrepancies in representativeness while recognising that this threshold is intended as a pragmatic interpretive guide rather than a formal cut-off. Comparisons were therefore interpreted by jointly considering statistical significance and the magnitude of observed differences. Statistical comparisons of means (ie, mean activity rates) were not performed owing to the lack of distributional information in external data sources.

### Patient and public involvement

Patients or members of the public were not involved in the design, conduct, reporting or dissemination plans of this research, as the study focused exclusively on methodological aspects of healthcare workforce data and comparisons with existing institutional and professional sources.

## Results

The data search identified 14 relevant sources of information on healthcare professionals in Switzerland, including scientific articles, federal statistics and national reports (table 1). These sources were heterogeneous in origin and design. Some, such as the Federal Office of Public Health statistics^21 22^ or the FMH physician statistics,^23^ were derived from administrative or association-based registers, whereas others, including SHURP^24^ and the occupational therapy workforce survey,^25^ were based on voluntary surveys with varying response rates. The scope of these data sources also varied: while some covered entire professions at the national level, others were restricted to specific care settings such as hospitals, nursing homes or home care. Accordingly, sources differed in their similarity to the SCOHPICA-HCP target population and in their estimated representativeness. In total, we identified comparable information for 10 professions or professional groups: physicians, pharmacists, registered nurses, auxiliary nursing staff (eg, care assistants, nursing assistants and healthcare assistants), intermediate nursing staff (eg, certified health and social care assistants and certified nursing assistants), midwives, dietitians, occupational therapists, paramedics and physiotherapists. Table 1 summarises the main characteristics of each source, including year and professions covered. Given these differences in scope, content and data collection methods, observed discrepancies between SCOHPICA-HCP and external sources may reflect not only genuine population discrepancies but also methodological limitations.

**Table 1:** Available data sources in Switzerland

Name and reference of the data source	Year	Healthcare professionals included in the present study	Type of data	Label*	Estimated target-population representativeness†	Scope similarity to the SCOHPICA-HCP population‡
Swiss Health Observatory (Obsan) - primary care physicians in Switzerland^29^	2023	Physicians	Institutional report	Obsan physicians 2023	3	3
Swiss Medical Association (FMH) - physician statistics^23^	2023	Physicians	Professional association data	FMH 2023	4	5
Federal Office of Public Health (FOPH) - physician statistics^21^	2023	Physicians	Administrative data	FOPH physicians 2023	5	5
FOPH - pharmacist statistics^22^	2023	Pharmacists	Administrative data	FOPH pharmacists 2023	5	5
Obsan - future outpatient primary care^30^	2023	Pharmacists and physiotherapists	Institutional report	Obsan outpatient 2023	3	3
Swiss Physiotherapists Association (Physioswiss) report^31^	2019	Physiotherapists	Professional association data	Physioswiss 2019	4	5
Federal Statistical Office (FSO) - hospital statistics (KS)§	2019	Registered nurses, auxiliary nursing staff, intermediate nursing staff, midwives, dietitians, occupational therapists and paramedics	Administrative data	FSO hospital 2019	5	4
FSO - Statistics of socio-medical institutions/nursing homes (SOMED)§	2016	Registered nurses, auxiliary nursing staff and intermediate nursing staff	Administrative data	FSO SOMED 2016	5	3
FSO - home care and support statistics (SPITEX) and structural survey §	2021	Registered nurses, auxiliary nursing staff and intermediate nursing staff	Administrative data	FSO SPITEX 2021	5	3
Swiss Nursing Home Human Resources Project (SHURP) - final report^24^	2018	Registered nurses	Research study	SHURP 2018	3	3
Mahlstein *et al* - professional statistics on dietitians in Switzerland^32^	2017	Dietitians	Institutional report	Mahlstein 2017	3	5
Ballmer *et al* - size and structure of the Swiss occupational therapy workforce^25^	2023	Occupational therapists	Research study	Ballmer 2023	3	5
Obsan - rescue services in Switzerland^33^	2017	Paramedics	Institutional report	Obsan paramedics 2017	4	5
Swiss Paramedic Association (Swiss rescue) statistics^34^	2023	Paramedics	Professional association data	Swiss rescue 2023	4	5

Note: Full references are available in the reference list.

* The labels are used in tables2^4^.

† Target-population representativeness scale: 5 = exhaustive administrative data; 4 = broad register or professional association statistics; 3 = large voluntary survey; 2 = moderate sample, participation concerns; 1 = small or selective sample.

‡ Similarity-of-scope scale: 5 = national, all settings; 4 = national, covering multiple settings but not all; 3 = national, restricted to specific settings; 2 = subnational or institution-based population; 1 = highly specific subgroup.

§ Available through the report: Swiss Health Observatory - Health workforce in Switzerland.^35^

SCOHPICA-HCP, Swiss Cohort of Healthcare Professionals and Informal Caregivers - Healthcare Professionals.

**Table 2:** Characteristics of physicians and pharmacists

Physicians				
	Obsan physicians 2023 (n=1114)	FMH 2023 (n=41 100)	FOPH physicians 2023 (n=39 780)	SCOHPICA Baseline 2022 to 2023 (n=715)
Female	45.9%*†	47.0%*	43.0%*†	57.0%
Age (years)				
Mean age	NA	50	54	45
≤45‡	22.5%*†	36.0%*†	28.0%*†	53.4%
46–55‡	29.1%	26.3%	28.0%	27.7%
56–65‡	31.2%*†	24.9%*	26.0%*	16.0%
>65‡	17.2%*†	12.2%*	17.0%*†	2.9%
Mean activity rate (in %)	NA	80.0	NA	86.2
Number of hours of work/week				
<35	26.2%*†	NA	NA	15.9%
35–44	23.4%	NA	NA	23.1%
≥45	50.2%*†	NA	NA	61.0%
Specialty				
Internal general medicine	72.3%*†	21.0%*†	NA	43.6%
Psychiatry	NA	9.7%	NA	9.2%
Paediatrics	15.8%*	5.3%*	NA	8.5%
Linguistic distribution				
German	68.2%*†	69.1%*†	NA	36.1%
French	26.7%*†	26.3%*†	NA	59.9%
Italian	5.1%	4.6%	NA	4.0%

Pharmacists			
	FOPH pharmacists 2023 (n=7183)	Obsan outpatient 2023 (n=720)	SCOHPICA Baseline 2022 to 2023 (n=156)
Female	74.0%	74.3%	70.5%
Age (years)			
Mean age	50	NA	43
≤45	40.0%*†	48.2%*†	62.9%
>46	60.0%*†	51.8%*†	37.0%
Degree-issuing country			
Swiss diploma	76.0%	NA	74.2%
Foreign diploma	24.0%	NA	25.8%
Linguistic distribution			
German	NA	56.3%*†	37.2%
French	NA	41.3%*†	52.6%
Italian	NA	2.0%*	10.2%

Note: Labels for the data sources are taken from table 1, which presents each source’s characteristics.

* p<0.05 for a two-proportion z-test between the external data source and the SCOHPICA-HCP sample.

† Absolute difference >10% compared with SCOHPICA-HCP.

‡ The age groups have been adjusted for greater clarity, with a difference of ±1 year depending on the studies being compared.

FMH, Swiss Medical Association ; FOPH, Federal Office of Public Health; Obsan, Swiss Health Observatory; SCOHPICA, Swiss Cohort of Healthcare Professionals and Informal Caregivers; SCOHPICA-HCP, Swiss Cohort of Healthcare Professionals and Informal Caregivers - Healthcare Professionals .

**Table 3:** Characteristics of nursing and care professions

Registered nurses					
	FSO hospital 2019 (n=57 750)	FSO SOMED 2016 (n=20058)	FSO SPITEX 2021 (n=12 824)	SHURP 2018 (n=4442)	SCOHPICA Baseline 2022–2023 (n=1753)
Female	84.1%	85.6%	NA	88.7%*	84.8%
Age (years)					
<35‡	36.1%*	21.6%*†	NA	37.3%*	32.1%
35−44‡	25.3%	21.6%*	NA	17.7%*	27.0%
45−54‡	22.3%	28.8%*	NA	25.5%	23.2%
≥55‡	16.3%	28.0%*†	NA	19.6%	17.5%
Mean activity rate (in %)	75.4	74.5	NA	80.0	79.4
Sector of practice					
Psychiatry	12.7%*	NA	NA	NA	14.7%
Rehabilitation and geriatrics	7.2%	NA	NA	NA	7.1%
Degree-issuing country					
Swiss diploma	NA	NA	NA	84.4%*†	72.2%
Place of training					
Training abroad	NA	28.9%	NA	NA	27.8%
Linguistic distribution					
German	67.4%*†	75.7%*†	64.2%*†	NA	43.1%
French	28.0%*†	19.2%*†	28.8%*†	NA	51.9%
Italian	4.6%	5.1%	7.0%*	NA	5.0%

Auxiliary nursing staff				
	FSO hospital 2019 (n=5963)	FSO SOMED 2019 (n=10 541)	FSO SPITEX 2021 (n=2342)	SCOHPICA Baseline 2022–2023 (n=156)
Female	84.9%	87.6%	NA	88.9%
Age (years)				
<35	26.5%*	36.7%*†	NA	16.5%
35−44	19.3%	19.9%	NA	22.3%
45−54	28.2%	23.7%*	NA	31.7%
≥55	26.0%	19.7%*	NA	29.5%
Mean activity rate (in %)	77.7	76.6	NA	75.0
Sector of practice				
Psychiatry	6.1%	NA	NA	9.1%
Rehabilitation and geriatrics	18.8%*†	NA	NA	5.9%
Linguistic distribution				
German	49.3%*†	56.1%*†	49.1%*†	14.1%
French	43.5%*†	29.4%*†	39.0%*†	84.6%
Italian	7.3%*	14.5%*†	11.9%*†	1.3%

Intermediate nursing staff				
	FSO hospital 2019 (n=11 280)	FSO SOMED 2019 (n=20 638)	FSO SPITEX 2021 (n=8171)	SCOHPICA Baseline 2022–2023 (n=215)
Female	87.6%	88.5%	NA	87.0%
Age (years)				
<35	69.5%*†	52.4%*†	NA	41.1%
35−44	10.9%*†	13.2%*†	NA	24.5%
45−54	11.3%*†	17.8%	NA	22.0%
≥55	8.3%*	16.6%	NA	12.3%
Mean activity rate (in %)	80.9	75.4	NA	78.6
Sector of practice				
Psychiatry	12.9%	NA	NA	13.5%
Rehabilitation and geriatrics	13.5%	NA	NA	9.8%
Linguistic distribution				
German	80.1%*†	79.6%*†	70.7%*†	46.5%
French	18.8%*†	16.9%*†	24.1%*†	50.7%
Italian	0.6%*	3.6%	5.2%	2.8%

Midwives		
	FSO hospital 2019 (n=2849)	SCOHPICA Baseline 2022–2023 (n=440)
Female	99.0%	98.2%
Age ≥50 (years)	37.0%*†	21.9%
Mean activity rate (in %)	68.3	76.3
Linguistic distribution		
German	65.0%	70.4%
French	32.2%	28.2%
Italian	2.8%	1.4%

Note: Labels for the data sources are taken from table 1, which presents each source’s characteristics.

* p<0.05 for a two-proportion z-test between the external data source and the SCOHPICA-HCP sample.

† Absolute difference >10% compared with SCOHPICA-HCP.

‡ The age groups have been adjusted for greater clarity, with a difference of ±1 year depending on the studies being compared.

FSO, Federal Statistical Office; SCOHPICA, Swiss Cohort of Healthcare Professionals and Informal Caregivers ; SCOHPICA-HCP, Swiss Cohort of Healthcare Professionals and Informal Caregivers - Healthcare Professionals; SHURP, Swiss Nursing Home Human Resources Project; SOMED, statistics of socio-medical institutions/nursing homes; SPITEX, home care and support statistics.

**Table 4:** Characteristics and comparison of allied health professionals

Dietitians			
	Mahlstein 2017 (n=756)	FSO hospital 2019 (n=864)	SCOHPICA Baseline 2022–2023 (n=93)
Female	95.0%	NA	92.4%
Activity rate			
80%–100%	33.0%*†	NA	58.4%
60%–80%	27.0%	NA	32.6%
<60%	40.0%*†	NA	9.0%
Sector of practice			
Psychiatry	NA	2.8%*†	18.3%
Rehabilitation and geriatrics	NA	9.4%*†	28.0%
Linguistic distribution			
German	NA	69.2%*†	18.3%
French	NA	30.1%*†	79.6%
Italian	NA	0.0%*	2.1%

Occupational therapists			
	Ballmer 2023 (n=968)	FSO hospital 2019 (n=1501)	SCOHPICA Baseline 2022–2023 (n=238)
Female	90.1%	NA	93.0%
Age (years)			
Mean age	48.1	NA	37.3
<30	27.3%	NA	27.6%
30–39	35.8%	NA	39.5%
40–49	20.0%	NA	16.7%
50–59	13.8%	NA	13.6%
≥60	3.2%	NA	2.6%
Mean activity rate (in %)	67.9	NA	78.0
Sector of practice			
Psychiatry	NA	19.6%	22.3%
Rehabilitation and geriatrics	NA	26.0%*†	66.4%
Degree-issuing country			
Swiss diploma	65.5%*	64.5%*†	75.0%
Linguistic distribution			
German	61.5%*†	71.2%*†	41.6%
French	34.5%*†	26.5%*†	54.6%
Italian	4.0%	2.3%	3.8%

Paramedics				
	Obsan paramedics 2017 (n=3700)	Swiss rescue 2023 (n=3653)	FSO hospital 2019 (n=932)	SCOHPICA Baseline 2022–2023 (n=349)
Female	30.0%*	38.7%	NA	36.8%
Age (years)				
< 35	37.0%	33.8%*	NA	41.7%
35–49	47.0%	47.2%	NA	43.1%
≥50	16.0%	19.0%	NA	15.2%
Mean activity rate (in %)	NA	79.0	NA	93.1
Linguistic distribution				
German	NA	NA	88.3%*†	39.8%
French	NA	NA	11.7%*†	44.4%
Italian	NA	NA	0.0%*†	15.8%

Physiotherapists			
	Physioswiss 2019 (n=9315)	Obsan outpatient 2023 (n=1290)	SCOHPICA Baseline 2022–2023 (n=463)
Female	NA	78.4%	77.3%
Age (years)			
≤30	NA	16.0%*	20.4%
31–45	NA	39.7%*	47.9%
≥46	NA	44.3%*†	31.7%
Linguistic distribution			
German	72.0%*	89.1%*†	58.3%
French	23.0%*	10.4%*†	36.3%
Italian	5.0%	0.5%*	5.4%

Note: Labels for the data sources are taken from table 1, which presents each source’s characteristics.

* p<0.05 for a two-proportion z-test between the external data source and the SCOHPICA-HCP sample.

† Absolute difference>10% compared with SCOHPICA-HCP.

FSO, Federal Statistical Office; SCOHPICA, Swiss Cohort of Healthcare Professionals and Informal Caregivers; SCOHPICA-HCP, Swiss Cohort of Healthcare Professionals and Informal Caregivers - Healthcare Professionals.

Tables2^4^ present the details of all available data sources, as well as the characteristics of the SCOHPICA-HCP participants, by profession. Hereafter, we highlight differences that were statistically significant and/or exceeded the 10% guide used to assess potentially meaningful discrepancies.

Among the socio-demographic variables, age, sex and spoken language were the most often reported in the data sources considered. SCOHPICA-HCP participants tended to be younger than those in the comparative data sources for physicians, pharmacists, paramedics and physiotherapists, with several differences being statistically significant and exceeding the predefined magnitude threshold, particularly for physicians and pharmacists. A contrasting picture was noted for nursing and care professions (ie, registered nurses, auxiliary nursing staff, intermediate nursing staff and midwives), with SCOHPICA-HCP participants being sometimes younger, sometimes older, depending on the comparative source and professional group considered. No statistically significant difference was observed in the age categories of occupational therapists. Regarding sex, we observed a similar representation of female participants in SCOHPICA-HCP compared with the available data for nursing and care professions and for different allied health professions, with most differences remaining below 10 percentage points. However, there was a higher proportion of women among physicians in SCOHPICA-HCP compared with other data sources. Concerning spoken language, there was an over-representation of French speakers and an under-representation of German speakers for all professions in SCOHPICA-HCP compared with existing data, except for midwives; these differences were often both statistically significant and above the predefined threshold for potentially meaningful differences.

Regarding the socio-professional variables, only the activity rate was frequently reported by comparative data sources. The mean activity rate was broadly comparable between SCOHPICA-HCP and other data sources for most professions, with somewhat higher descriptive values observed in SCOHPICA-HCP for physicians, midwives, occupational therapists and paramedics. However, only 9% of dietitians in SCOHPICA-HCP indicated working at a rate lower than 60%, whereas 40% did so in the comparative source (table 4).

Regarding the socio-demographic and professional characteristics that were retrieved for specific professions, the rate of completing a diploma programme in Switzerland was similar for pharmacists in SCOHPICA-HCP compared with other data sources but lower for registered nurses and higher for occupational therapists. Comparisons for physicians indicated that SCOHPICA-HCP participants reported working more hours per week (table 2). Finally, comparisons by sector of practice for nursing and care professionals, dietitians and occupational therapists showed inconsistent patterns across data sources and were limited by differences in the populations covered (eg, hospital-based vs all practice settings). Similar constraints applied to comparisons of specialty for physicians.

## Discussion

Overall, the combined interpretation of statistical significance and absolute differences suggested that healthcare professionals participating in SCOHPICA were generally comparable to those from other available Swiss data sources for some characteristics, particularly sex distribution. The most consistent discrepancies concerned age and linguistic distribution. SCOHPICA-HCP participants tended to be younger than those in several comparative data sources, particularly among physicians and pharmacists, while French speakers were over-represented and German speakers under-represented across most professions. Several statistically significant differences were limited in magnitude and should be interpreted cautiously, whereas those also meaningful in magnitude were more likely to indicate substantive divergences between SCOHPICA-HCP and external data sources. For socio-professional characteristics, no clear overall pattern emerged, as the relevant variables and sub-populations varied considerably across sources. Notably, few comparative sources combined both high similarity-of-scope and high target-population representativeness, underscoring the challenges of identifying suitable reference data for healthcare workforce studies in Switzerland. Based on the available sources and within the limits of this study, participants from certain professions in SCOHPICA-HCP therefore appear to differ from healthcare professionals practising in Switzerland for some of the compared variables, which may be explained by several reasons discussed hereafter.

Concerning age differences, we can explain the apparent overrepresentation of younger individuals in SCOHPICA-HCP by the exclusive use of an online data collection method. Indeed, a similar pattern was found in the PASTA project, where opportunistic recruitment was combined with a web-based survey instead of more traditional recruitment approaches.^6^ Another hypothesis is that younger people may be more inclined to participate in surveys concerning their working conditions, in the hope that meaningful changes would occur while they are still within an age group actively engaged in the workforce. The over-representation of French speakers in SCOHPICA-HCP can reasonably be attributed to the fact that the study is managed from the French-speaking part of Switzerland.^14^ However, despite this potentially meaningful linguistic difference, several other characteristics remained broadly comparable to existing national data. This suggests that participating healthcare professionals do not necessarily differ in terms of their characteristics across Swiss regions. Furthermore, this difference should be interpreted with caution, as the linguistic variable is defined by the language chosen to fill in SCOHPICA-HCP’s questionnaire, while it was not always specified for the other data sources. Therefore, it does not entirely reflect the geographical distribution of participants, especially since healthcare professionals can easily move across the country, which mitigates the relevance of this distinction.

When discussing representativeness, it is important to distinguish between representativeness in terms of estimation, such as a measured treatment effect, or in terms of interpretation, such as the direction or meaning of the effect.^1^ Generalisation in estimation of study results to the target population depends on the study design, particularly whether the sample was selected randomly or if effect modifiers were accounted for. Conversely, generalisation in interpretation relies more on the underlying scientific validity, without necessarily considering random sampling or the existence of effect modifiers. In this study, we are primarily discussing generalisation in interpretation because of the recruitment method used. The data collected in SCOHPICA can be regarded by any standard as rich and comprehensive.^26^ This extensive richness of data represents an advantage of cohort studies compared with registers, which typically collect a limited number of variables that often differ between registers, thereby complicating meaningful comparisons. SCOHPICA-HCP data reveals a great diversity, both intra- and inter-professional, suggesting that the studied sample represents a broad range of healthcare professionals practising in Switzerland. Consequently, data from SCOHPICA-HCP provide a generally accurate overview of the composition of the healthcare workforce in Switzerland while offering opportunities for in-depth analysis of the dynamics and challenges specific to each professional group within the Swiss healthcare system.

To increase the representativeness of participating professionals and enable improved monitoring, management and planning of the healthcare workforce, comprehensive registers are necessary, with each professional required to regularly update their information. Some countries, such as Canada, aim to advance the implementation of such national registers.^27^ However, important challenges remain. Data are usually collected by different regions, professions and institutions using heterogeneous definitions and formats, limiting comparability. To be fit for purpose, national registers require a minimum set of standardised, comparable core data elements on healthcare professionals.^28^ Furthermore, continuous updating may create an administrative burden for professionals and requires appropriate governance, technical infrastructure and sustainable resources. As career paths evolve over time, including changes in employment status, working hours or specialisation, one-time snapshots have limited value. Continuous tracking would provide a more accurate and dynamic overview of the healthcare workforce, particularly in decentralised systems such as Switzerland, but requires balancing data completeness with feasibility and acceptability.

To illustrate the difficulty of reaching healthcare professionals, we provide the following example: during the 2024 fall recruitment of SCOHPICA, we sent letters to a random sample of 14 691 addresses derived from two partial registers of non-university health professions (ie, NAREG and GesReg) as an alternative recruitment method.^12 13^ Out of these 14 691 letters, 4972 were returned to us due to undeliverable addresses, which represents 33.2% of outdated data. This highlights the issue with such incomplete registers that only contain the year of birth, graduation date and professional mailing address of healthcare professionals. First, sending physical mail is costly and significantly complicates efforts to contact these professionals. Second, the population of healthcare professionals is highly mobile, and if addresses are not automatically updated, a high rate of returned mail can be expected when using postal delivery.

The main strength of this study is that it is an innovative project that gathered, for the first time, important data on healthcare professionals practising in Switzerland, from various and diverse sources. This allowed us to compare socio-demographic and socio-professional characteristics to those of SCOHPICA-HCP participants and assess their representativeness; it can also serve as a foundation for any future studies on this population.

Nevertheless, this study presents several limitations. The available comparative data were not exhaustive, and no comprehensive, up-to-date national register currently contains socio-demographic and socio-professional information on all healthcare professionals practising in Switzerland. We therefore compared SCOHPICA-HCP participants with the best available data, although these cannot be considered true reference data. We included as many relevant comparative sources as possible to strengthen the assessment; however, their representativeness cannot be guaranteed, as they were not all derived from random or exhaustive samples. Moreover, their suitability for representing the healthcare workforce was limited by the unavailability of some variables, such as employment status (eg, self-employed vs salaried), years of professional experience or family characteristics.

Another limitation relates to the heterogeneity of data collection methods, measured variables and target populations across comparative sources. These differences made direct comparisons challenging, as variables were not always defined or categorised in the same way, for example, for age categories. In addition, some characteristics, such as activity rate, were self-reported in SCOHPICA-HCP but derived from administrative sources or differently defined in several comparative datasets, which may have contributed to inconsistencies. Comparisons for certain characteristics, such as sector of practice and physicians’ specialty, also yielded inconsistent findings because some external sources were restricted to specific settings (eg, hospital-based professionals) or categories (eg, board-certified physicians), whereas SCOHPICA-HCP included healthcare professionals across all practice settings, irrespective of postgraduate training level or employment status.

A further limitation concerns the comparison methods themselves. Statistical testing helped identify differences between SCOHPICA-HCP and external data sources, but p-values are influenced by sample size, and the large number of comparisons performed increases the risk of false-positive findings. Conversely, the predefined magnitude threshold was pragmatic and necessarily somewhat arbitrary and should be interpreted as an aid to interpretation rather than as a definitive criterion for representativeness. Combining statistical testing with an assessment of absolute differences therefore provided a more nuanced interpretation of discrepancies between SCOHPICA-HCP and external data sources.

### Conclusions

This study illustrates the challenges and opportunities of assessing representativeness in healthcare workforce research when comprehensive and up-to-date registers are not available. Using SCOHPICA-HCP as a case study, it is possible to conduct indirect recruitment while obtaining results that can be generalised in interpretation to the healthcare professionals’ population under study, provided that the limitations of this approach are sufficiently emphasised. The methodological issues encountered—fragmented data sources, outdated registers and variability in indicators—mirror those reported in other countries. Consequently, the lessons from this study extend beyond Switzerland: they underscore the need for internationally comparable strategies to monitor health workforces, whether through improved national registers or innovative cohort designs. By documenting both the strengths and limitations of non-probability recruitment, this article provides guidance for researchers and policymakers seeking to balance feasibility with representativeness in healthcare workforce studies worldwide.

## Data Availability

Data are available upon reasonable request.

## References

[1] Rudolph JE, Zhong Y, Duggal P (2023). Defining representativeness of study samples in medical and population health research. *BMJ Med*.

[2] Abel KM, Radojčić MR, Rayner A (2023). Representativeness in health research studies: an audit of Greater Manchester Clinical Research Network studies between 2016 and 2021. BMC Med.

[3] Kalton G (2020). Introduction to survey sampling.

[4] Flanigan TS, McFarlane E, Cook S (2008). Conducting survey research among physicians and other medical professionals: a review of current literature. Proceedings of the Survey Research Methods Section, American Statistical Association.

[5] Vehovar V, Toepoel V, Steinmetz S, Wolf C, Joye D, Smith TW (2016). The SAGE handbook of survey methodology.

[6] Gaupp-Berghausen M, Raser E, Anaya-Boig E (2019). Evaluation of Different Recruitment Methods: Longitudinal, Web-Based, Pan-European Physical Activity Through Sustainable Transport Approaches (PASTA) Project. *J Med Internet Res*.

[7] Kim KS (2022). Methodology of Non-probability Sampling in Survey Research. *AJBSR*.

[8] Wiśniowski A, Sakshaug JW, Perez Ruiz DA (2020). Integrating Probability and Nonprobability Samples for Survey Inference. J Surv Stat Methodol.

[9] Karvanen J, Tolonen H, Härkänen T (2016). Selection bias was reduced by recontacting nonparticipants. J Clin Epidemiol.

[10] Federal Office of Public Health (FOPH) Register of medical professions (MedReg), Bern. https://www.bag.admin.ch/fr/registre-des-professions-medicales-medreg.

[11] National Register of Healthcare Professions (NAREG). Bern: GDK/CDS Swiss Conference of the Cantonal Ministers of Public Health; Swiss Red Cross. https://www.nareg.ch.

[12] Swiss Conference of the Cantonal Ministers of Public Health Health Professions Register (GesReg). Bern: GDK/CDS. https://www.gesreg.ch.

[13] Zhao Y, Li X, Leckcivilize A (2025). Longitudinal tracking of healthcare professionals: a methodological scoping review. BMC Med Res Methodol.

[14] Peytremann-Bridevaux I, Jolidon V, Jubin J (2024). Protocol for the Swiss COhort of Healthcare Professionals and Informal CAregivers (SCOHPICA): Professional trajectories, intention to stay in or leave the job and well-being of healthcare professionals. PLoS One.

[15] Peytremann-Bridevaux I, Oulevey Bachmann A, Gilles I (2024). The Swiss Cohort of Healthcare Professionals and Informal Caregivers (SCOHPICA): Datasets. Lausanne (Switzerland): Center for Primary Care and Public Health (Unisanté), University of Lausanne; 2024. Version 1.0.

[16] Arksey H, O’Malley L (2005). Scoping studies: towards a methodological framework. Int J Soc Res Methodol.

[17] Swiss Federal Statistical Office (FSO) Structural survey (relevé structurel). Neuchâtel. https://www.bfs.admin.ch/bfs/fr/home/bases-statistiques/recensement-population/quatre-elements-cles/releve-structurel.html.

[18] Swiss Federal Statistical Office (FSO) Swiss Labour Force Survey (SLFS / ESPA), Neuchâtel. https://www.bfs.admin.ch/bfs/fr/home/statistiques/travail-remuneration/enquetes/espa.html.

[19] Tricco AC, Lillie E, Zarin W (2018). PRISMA Extension for Scoping Reviews (PRISMA-ScR): Checklist and Explanation. Ann Intern Med.

[20] Peter KA, Voirol C, Kunz S (2024). Factors associated with health professionals’ stress reactions, job satisfaction, intention to leave and health-related outcomes in acute care, rehabilitation and psychiatric hospitals, nursing homes and home care organisations. BMC Health Serv Res.

[21] Federal Office of Public Health (FOPH) (2023). Statistiques médecins. Bern. https://www.bag.admin.ch/fr/statistiques-medecins.

[22] Federal Office of Public Health (FOPH) (2023). Statistiques pharmaciens. Bern. https://www.bag.admin.ch/fr/statistiques-pharmaciens.

[23] Swiss Medical Association (FMH) FMH-Ärztestatistik / Statistique médicale de la FMH 2023. https://aerztestatistik.fmh.ch.

[24] Zúñiga F, Favez L, Baumann S (2018). Étude SHURP 2018 – Rapport final Personnel et qualité des soins dans les établissements médico-sociaux en Suisse alémanique et en Suisse romande.

[25] Ballmer T, Kühne N, Petrig A (2023). The Size and Structure of the Swiss Occupational Therapy Workforce. A Survey Study / Anzahl und Struktur der Ergotherapie-Arbeitsplätze in der Schweiz: eine Online-Befragung. Int J Health Prof.

[26] Jolidon V, Jubin J, Zuercher E (2024). Health Workforce Challenges: Key Findings From the Swiss Cohort of Healthcare Professionals and Informal Caregivers (SCOHPICA). Int J Public Health.

[27] Leslie K, Demers C, Steinecke R (2022). Pan-Canadian Registration and Licensure of Health Professionals: A Path Forward Emerging from a Best Brains Exchange Policy Dialogue. hcpol.

[28] Bourgeault IL (2021). A path to improved health workforce planning, policy & management in Canada: The critical coordinating and convening roles for the federal government to play in addressing 8% of its GDP. SPP Publications.

[29] Swiss Health Observatory (Obsan) (2023). Ärztinnen und Ärzte in der Grundversorgung – Situation in der Schweiz und im internationalen Vergleich. Neuchâtel. https://www.obsan.admin.ch/de/publikationen/2023-aerztinnen-und-aerzte-der-grundversorgung-situation-der-schweiz-und-im.

[30] Brandt S, Essig S, Balthasar A (2023). Zukünftige ambulante Grundversorgung: Einstellungen und Präferenzen von Medizinal und Gesundheitsfachpersonen ausgewählter Berufsgruppen, Obsan Bericht. https://www.obsan.admin.ch/fr/publications/2023-zukunftige-ambulante-grundversorgung-einstellungen-und-praferenzen-von-medizinal.

[31] Physioswiss (2019). Rapport annuel 2019. Bern: Association suisse de physiothérapie. https://physioswiss.ch.

[32] Mahlstein A, Weishaupt E (2018). Berufsstatistik über die ernährungsberater und ernährungsberaterinnen in der schweiz - resultate 2017. berufsfeld, arbeits-, aus- und weiterbildungssituation von ernährungsberater und ernährungsberaterinnen. https://svde-asdd.ch/wp-content/uploads/2018/12/2018-10-31_Publikation_Berufsstatistik_2017.pdf.

[33] Swiss Health Observatory (Obsan) (2017). Les services de sauvetage en Suisse. Neuchâtel. https://www.obsan.admin.ch/fr/publications/2017-les-services-de-sauvetage-en-suisse.

[34] Swiss Paramedic Association (2023). Le sauvetage Suisse 2023 en chiffres. Swiss Rescue. https://www.144.ch/wp-content/uploads/2024/05/SPA_SOL_2-24_web_Das-Schweizer-Rettungswesen-in-Zahlen.pdf.

[35] Merçay C, Grünig A, Dolder P Personnel de santé en Suisse – Rapport national Obsan 2021. https://www.obsan.admin.ch/fr/publications/2021-personnel-de-sante-en-suisse-rapport-national-2021.

