# Supplementary material for: Indirect participant recruitment and representativeness in healthcare workforce research: lessons from a comparative study of the SCOHPICA cohort
Source: BMJ Open. 2026 Jun 28;16(6):e110324. doi: 10.1136/bmjopen-2025-110324 (PMC13311568; doi:10.1136/bmjopen-2025-110324)
Supplement: Supplementary file 1 [file bmjopen-2025-110324.reviewer_comments.pdf]

PEER REVIEW HISTORY

BMJ Open publishes all reviews undertaken for accepted manuscripts. Reviewers are asked to complete a checklist review form and are provided with free text boxes to elaborate on their assessment. These free text comments are reproduced below.

ARTICLE DETAILS

Title (Provisional)

Indirect Participant Recruitment and Representativeness in Healthcare Workforce Research: Lessons from a Comparative Study of the SCOHPICA Cohort

Authors

Hennard, Antoine; Roth, Leonard; Storari, Chiara; Zuercher, Emilie; Jubin, Jonathan; Oulevey Bachmann, Annie; Gilles, Ingrid; Peytremann-Bridevaux, Isabelle

VERSION 1 - REVIEW

**Reviewer**	**1**
**Name**	**Blake, Helen**
**Affiliation**	**London School of Hygiene & Tropical Medicine**
**Date**	**03-Nov-2025**
**COI**	**None**

Thank you for the opportunity to review this manuscript. The authors have undertaken an exploratory descriptive analysis that shows that participants included in their SCOHPICA study are broadly similar to participants in other Swiss datasets, except for age and language.

Abstract:

1. The abstract does not fully agree with what was done in the manuscript: the objective states that the manuscript will explore how indirect recruitment methods can be applied. But the details of the indirect recruitment for SCOHPICA are stated, without much consideration on alternative ways to apply indirect recruitment methods or key considerations. I recommend amending the objective to reflect that the manuscript focusses on assessing whether the study participants were representative.

2. Also, line 14 states that the data sources were collected between 2016 and 2024. But the earliest year in Table 1 is 2017. It would also be helpful to state that the SCOHPICA baseline sample was from the 2022-2023 recruitment waves.

Methods:

3. The Study Design section mentions recruitment sources for SCOHPICA. Is there a difference in participant characteristics by recruitment source? Can you compare subgroups in SCOHPICA with other data sources that have a similar recruitment method? E.g. Comparing SCOHPICA participants who were recruited through institutional partners to participants in data source that also recruited through institutional partners?

4. Line 130 describes the data search as 'comprehensive'. Can the authors demonstrate this, perhaps by referring to any best practice guidance? I am not familiar with Swiss data sources myself, but I was surprised that the Switzerland census did not seem to be mentioned. If the census included job type, I would think that this would be a good resource to use in order to assess generalisability of the SCOHPICA participants.

5. Line 155 mentions a 'group consensus' – please give more details (e.g. How many/who participated, how were disagreements settled, etc?)

Results:

6. I appreciate the discussion of the difference in measurement of specialty (lines 198-203), but think it would fit better in the limitations section of the Discussion. Please could you add more consideration of measurement differences for other variables, e.g. Is the information on hours worked per week self-reported in all datasets?

Discussion:

7. Lines 252-260 recommend having national registers with continuous tracking – please comment further on feasibility/challenges of this e.g. adding additional work burden to health care professionals.

8. The discussion notes that "the available comparative data is not exhaustive" (lines 276-277). Have the authors considered what supplemental information would be useful to have, if they were able to influence data collection? E.g. Whether workers are part-time or full-time?

9. Recalling the objective as currently written in the abstract, please discuss what other indirect recruitment methods could have been used, and why they were not used for SCOHPICA.

10. Limitation: it is possible that the characteristics of workers vary year by year. However, all comparisons are made to the 2022-2023 waves of SCOHPICA, whilst the years of the comparative datasets vary. Please discuss.

Tables:

11. Lines 47 and 162 in the manuscript say that 15 data sources were considered, but there only seems to be 13 listed in Table 1.

12. The names of the data sources in Table 1 does not seem to match up exactly to Tables 2-4 (e.g. I could not see FOPH in Table 1). It might help to assign labels to each data source in Table 1, and cite those labels in the headings of Tables 2-4.

13. If possible, it would be helpful to have notes in Table 1 describing how representative each data source is presumed to be (ideally as judged by impartial third party).

Minor comments:

14. There are lots of abbreviations in the main text that are not fully explained: e.g. PASTA (line 83), FMH (line 133), SHURP (line 135), STRAIN (line 136)

15. There are some odd phases/formatting: e.g. “physiotherapists were far less numerous to report being self-employed” (line 196). The word ‘caveat’ should not be in italics since it has been adopted into the English language (line 201).

16. Throughout the manuscript, the spelling “dietitian” is used, but Table 1 uses “dietician”.

**Reviewer**	**2**
**Name**	**Fowler-Davis, Sally**
**Affiliation**	**Anglia Ruskin University, Health, Medicine and Social Care**
**Date**	**27-Feb-2026**
**COI**	**None**

Thanks for allowing me to review this paper which is a small but important contribution to the overall study of healthcare workforces and has international relevance, given the difficulties monitoring recruitment, retention and wellbeing across health systems.

I found the paper easy to read and helpfully explained except in one respect- I thought that the term recruitment related to workforce recruitment practices not research recruitment in terms of sampling and participation. Could this be made clearer in the abstract and the title please.

**Reviewer**	**3**
**Name**	**Dawson, Jeremy**
**Affiliation**	**The University of Sheffield, Sheffield University Management School**
**Date**	**16-Mar-2026**
**COI**	**None**

Thank you for the opportunity to review this manuscript. Indirect recruitment of healthcare staff is indeed an important topic, and for that reason I think research in this area is welcome (although I was looking for more justification of why healthcare staff are different from others in this respect, and why learning from other sectors could not be applied to a greater extent than it is).

However, my main concern is that the methods used do not meet the objective. The manuscript presents a hypothesis - that participants recruited via these methods are similar enough to the general population for the research to be valid - but it does not then actually test this in a meaningful way, instead just presenting detailed descriptive statistics that indicate some similarity but without a clear justification for representativeness.

Rather, I would expect to see some more formal testing here. One way in which this might be done is to decide on appropriate tolerance levels for what consitutes similar samples (for example, within 10% of the popuation level on a particular characteristic - that is just off the top of my head though, and any figure used would need to be justified); and then presenting confidence intervals on the sample used to indicate whether the sample characteristic fall within these tolerances. If they do, then that is good evidence for representativeness. As it is, though, it is difficult to assess whether you have a statistically valid conclusion.

VERSION 1 - AUTHOR RESPONSE

Reviewer 1

Abstract:

1. The abstract does not fully agree with what was done in the manuscript: the objective states that the manuscript will explore how indirect recruitment methods can be applied. But the details of the indirect recruitment for SCOHPICA are stated, without much consideration on alternative ways to apply indirect recruitment methods or key considerations. I recommend amending the objective to reflect that the manuscript focusses on assessing whether the study participants were representative.

Thank you for correctly pointing out that the previous objectives did not fully reflect what was later discussed in the manuscript. We have revised the objectives accordingly (lines 3-4 and 111-114), such that they are now more tightly aligned with the study presented. The revised objectives read as follows: *"To assess the representativeness of participants recruited through indirect methods into a cohort of healthcare professionals in the absence of comprehensive and up-to-date national registers."*

2. Also, line 14 states that the data sources were collected between 2016 and 2024. But the earliest year in Table 1 is 2017. It would also be helpful to state that the SCOHPICA baseline sample was from the 2022-2023 recruitment waves.

Our apologies - Table 1 was indeed unclear, as in three cases it presented the report through which the data were available rather than the actual data sources. This table has been entirely reformatted, and it now shows the characteristics of all data sources, with the earliest year being in fact 2016. However, to ensure consistency across data sources, one source had to be removed (the justification for this is presented in lines 159-161).

Consequently, the latest year in Table 1 and in the rest of the manuscript (line 13 for instance) is now 2023.

Also, thank you for the suggestion concerning the SCOHPICA recruitment years. This has now been added in line 14 and the corresponding paragraph reads as follows: *"Healthcare professionals across 10 professional groups, with data drawn from 14 administrative, institutional, professional, and research sources collected between 2016 and 2023, alongside participants from the 2022-2023 baseline recruitment waves of the Swiss Cohort of Healthcare Professionals and Informal Caregivers (SCOHPICA)."*

Methods:

3. The Study Design section mentions recruitment sources for SCOHPICA. Is there a difference in participant characteristics by recruitment source? Can you compare subgroups in SCOHPICA with other data sources that have a similar recruitment method? E.g. Comparing SCOHPICA participants who were recruited through institutional partners to participants in data source that also recruited through institutional partners?

This is an interesting question. Unfortunately, we are not able answer it because we do not know how the SCOHPICA participants were specifically recruited, i.e., through which one of several recruitment methods each participant was reached. They may for instance have been contacted by their professional association, by their employer, or both (or by another mean). This makes comparisons between recruitment methods impossible, whether inside SCOHPICA or with the external data sources.

4. Line 130 describes the data search as 'comprehensive'. Can the authors demonstrate this, perhaps by referring to any best practice guidance? I am not familiar with Swiss data sources myself, but I was surprised that the Switzerland census did not seem to be mentioned. If the census included job type, I would think that this would be a good resource to use in order to assess generalisability of the SCOHPICA participants.

Thank you for challenging us to be more precise in how the data search was designed and conducted. This has now been clarified in greater detail. The methodological guidance for scoping reviews taken as inspiration ("Scoping studies: towards a methodological framework" by Arksey & O'Malley and "PRISMA extension for scoping reviews (PRISMA-ScR)" by Tricco et al.) is specified in lines 132-135 and 146-147. Furthermore, the reason why large-scale population surveys such as the national census could not be included is laid out in lines 139-142, stating in particular that the available information such as the professional category was not sufficiently detailed to be used as a comparative data source.

5. Line 155 mentions a 'group consensus' – please give more details (e.g. How many/who participated, how were disagreements settled, etc?)

Thank you for requesting further details on these comparisons. In the end, due to the third reviewer's comment, a different path was chosen, with statistical tests for the comparisons across professional groups and data sources instead of the original qualitative interpretation. The new method is described in lines 176-179 and the wording has been adapted accordingly at different places in the Results section.

Results:

6. I appreciate the discussion of the difference in measurement of specialty (lines 198-203), but think it would fit better in the limitations section of the Discussion. Please could you add more consideration of measurement differences for other variables, e.g. Is the information on hours worked per week self-reported in all datasets?

Thank you for this suggestion. We have kept a shortened and revised description of the mentioned results in lines 229-233. Parallelly, a third limitation was added in the discussion to elaborate further on this (lines 322-328). Finally, the second limitation was extended in lines 320-322 to account for the difference in measurement of a variable often reported (i.e., the activity rate). The current limitations paragraph (including the revised second and third limitations) now reads as follows:

"Nevertheless, our study presents three main limitations. The first is that the available comparative data are not exhaustive, and that there is no comprehensive and up-to-date national register containing socio-demographic or socio-professional information on all healthcare professionals practicing in Switzerland. We therefore compared SCOHPICA-HCP's participants with the best available data, although these are not strictly speaking reference data. We used as many relevant comparative sources as possible to increase reliability, although it cannot be guaranteed that these data are representative, as they do not originate from random samples either, making interpretation delicate. Moreover, the appropriateness of these data sources as representations of the healthcare workforce is limited by the unavailability of some variables, such as employment status (e.g., self-employed vs. salaried), years of experience in the profession, or family characteristics (e.g., having children or not). The second limitation is that the methods used to collect data generally differed across the comparative sources making comparisons challenging. The same applies to the measured variables, which often diverged (e.g., different age categories). In addition, some characteristics such as activity rate were self-reported in SCOHPICA-HCP but derived from administrative sources or differently defined in several comparative datasets, which may have contributed to inconsistencies and comparison difficulties. The third limitation is that comparisons by certain

characteristics, such as sector of practice and physicians' specialty, yielded inconsistent findings across data sources, partly due to differences in the target populations covered. For example, some external sources were restricted to specific settings (e.g., hospital-based professionals) or to certain categories (e.g., board-certified physicians), whereas SCOHPICA-HCP included healthcare professionals across all practice settings, irrespective of their post-graduate training level or employment status."

Discussion:

7. Lines 252-260 recommend having national registers with continuous tracking – please comment further on feasibility/challenges of this e.g. adding additional work burden to health care professionals.

Thank you for this suggestion. The discussion around the need for comprehensive registers has been revised to mention other important challenges and feasibility considerations (lines 287-292). It now reads as follows:

"Furthermore, continuous updating may create administrative burden for professionals and requires appropriate governance, technical infrastructure, and sustainable resources. As career paths evolve over time – including changes in employment status, working hours, or specialisation – one-time snapshots are of limited value. Continuous tracking would provide a more accurate and dynamic overview of the healthcare workforce, particularly in decentralised systems such as Switzerland, but requires balancing data completeness with feasibility and acceptability."

8. The discussion notes that "the available comparative data is not exhaustive" (lines 276-277). Have the authors considered what supplemental information would be useful to have, if they were able to influence data collection? E.g. Whether workers are part-time or full-time?

Thank you for this suggestion. We have revised the first limitation, which discusses the suitability of the external data sources, to reflect on what type of supplemental information would be useful for our comparative analysis as well as for other studies on the healthcare workforce (lines 314-317).

9. Recalling the objective as currently written in the abstract, please discuss what other indirect recruitment methods could have been used, and why they were not used for SCOHPICA.

Thank you for raising again this inconsistency regarding the objective of our study. As mentioned in our response to the first comment, the objective has been revised to reflect

what was actually performed and no longer focuses on indirect recruitment methods. In fact, all indirect recruitment methods that we could think of, were proposed by the entities contacted, and were feasible have been leveraged in the SCOHPICA project. The corresponding list has been slightly extended in line 122 to reflect this. The main potential reason why some recruitment channels could not be fully used is that not all the entities we contacted agreed to be involved to the same degree, which limited the effectiveness of certain channels.

10. Limitation: it is possible that the characteristics of workers vary year by year. However, all comparisons are made to the 2022-2023 waves of SCOHPICA, whilst the years of the comparative datasets vary. Please discuss.

The reviewer is right to note that the original manuscript did not provide sufficient explanation for the range of data collection years. The revised manuscript addresses this question in lines 153-157. In essence, the general structure of the healthcare workforce is not expected to vary substantially from year to year; as a side note, this assumption is at least partially supported by the observed stability of the newly recruited SCOHPICA-HCP participants' characteristics over time, as apparent on the project's open dashboard (available in French at https://scohpica.ch/professionnels-de-la-sante/resultats/). Nevertheless, we still decided to impose a lower bound on the sources' data collection years to limit the risk of relying on information that is too temporally distant.

Tables:

11. Lines 47 and 162 in the manuscript say that 15 data sources were considered, but there only seems to be 13 listed in Table 1.

This inconsistency was due to the way Table 1 was designed, as mentioned in our response to the second comment, with three of the data sources noted as remark in the table; this has been reformatted in the revised manuscript. Furthermore, one data source was excluded during revision, as explained in lines 159-161. There are thus 14 external data sources considered in the end, as stated in lines 12 and 187.

12. The names of the data sources in Table 1 does not seem to match up exactly to Tables 2-4 (e.g. I could not see FOPH in Table 1). It might help to assign labels to each data source in Table 1, and cite those labels in the headings of Tables 2-4.

Thank you for raising this issue. The names have been harmonised everywhere in the document. Moreover, we have followed the reviewer's suggestion and assigned a label to each data source in Table 1 (with the author, profession/setting where required, and year), which is then used in the other tables.

13. If possible, it would be helpful to have notes in Table 1 describing how representative each data source is presumed to be (ideally as judged by impartial third party).

Thank you for this suggestion. We addressed this in two steps in the revised manuscript. First, we assigned a rating to each comparative source describing its estimated target-population representativeness. Then, another rating was assigned to each source describing its similarity-of-scope to the SCOHPICA-HCP population. This process is described in the Methods (lines 167-174) and then briefly commented in the Results (lines 194-195) and in the Discussion (lines 242-244). These ratings appear in two separate columns in Table 1, with the scales specifically designed for this task presented in the notes. Three of the authors (including the two co-first) participated in this process. Disagreements were discussed together, and final decisions were made by majority vote.

Minor comments:

14. There are lots of abbreviations in the main text that are not fully explained: e.g. PASTA (line 83), FMH (line 133), SHURP (line 135), STRAIN (line 136)

Thank you for raising this. The abbreviations have now been harmonised throughout the manuscript (e.g., lines 87-88 and 137-139), as well as in the list of abbreviations at the end.

15. There are some odd phases/formatting: e.g. “physiotherapists were far less numerous to report being self-employed” (line 196). The word ‘caveat’ should not be in italics since it has been adopted into the English language (line 201).

The revised manuscript has been fully edited to avoid sentences that could be unclear to the reader.

16. Throughout the manuscript, the spelling “dietitian” is used, but Table 1 uses “dietician”.

Thank you for highlighting this typo and for the many helpful comments!

Reviewer 2

I found the paper easy to read and helpfully explained except in one respect- I thought that the term recruitment related to workforce recruitment practices not research recruitment in terms of sampling and participation. Could this be made clearer in the abstract and the title please.

Thank you for the kind comment and the relevant suggestion. This has been revised in the title and the abstract (line 26).

Reviewer 3

Thank you for the opportunity to review this manuscript. Indirect recruitment of healthcare staff is indeed an important topic, and for that reason I think research in this area is welcome (although I was looking for more justification of why healthcare staff are different from others in this respect, and why learning from other sectors could not be applied to a greater extent than it is).

Thank you for the encouraging words. We have added some considerations on the particularities of healthcare professionals in the Background section (lines 75-78). In fact, we do not argue that our situation is unique, nor that similar questions do not arise elsewhere. Rather, we underline that the healthcare workforce encompasses a heterogeneous set of professions and care settings, while also sharing a common purpose and being shaped by comparable working conditions and professional experiences. This combination of diversity and shared characteristics makes it a relevant case for examining issues of representativeness in a study such as ours.

However, my main concern is that the methods used do not meet the objective. The manuscript presents a hypothesis - that participants recruited via these methods are similar enough to the general population for the research to be valid - but it does not then actually test this in a meaningful way, instead just presenting detailed descriptive statistics that indicate some similarity but without a clear justification for representativeness.
Rather, I would expect to see some more formal testing here. One way in which this might be done is to decide on appropriate tolerance levels for what consitutes similar samples (for example, within 10% of the popuation level on a particular characteristic - that is just off the top of my head though, and any figure used would need to be justified); and then presenting confidence intervals on the sample used to indicate whether the sample characteristic fall within these tolerances. If they do, then that is good evidence for representativeness. As it is, though, it is difficult to assess whether you have a statistically valid conclusion.

Thank you for this challenging remark. Our original strategy was to assess representativeness through a qualitative interpretation of the differences, but we recognise that this approach may not be fully appropriate in this situation. We have thus followed the reviewer's recommendation and implemented a systematic statistical testing procedure for the comparisons between external data sources and the SCOHPICA sample. This procedure replaces the previous approach and is described in the Methods section (lines 176-179), where the corresponding paragraph reads as follows:

"Descriptive statistics were produced for each extracted variable within each professional group. Furthermore, proportions for each category of the variables were compared between external data sources and the SCOHPICA-HCP sample using two-proportion z-tests (two-sided), with statistical significance set at the 95% confidence level. Comparisons of means (i.e., mean activity rates) were not performed due to the lack of distributional information in the external data sources."

Consequently, Tables 2, 3, and 4 have been updated to show whether the differences in proportions observed between the external sources and our sample for each professional group were statistically significant. This is highlighted in the tables' notes. The conclusions do not vary much compared to the previous version of the manuscript (we refer the reviewer to the first paragraph of the discussion in lines 236-247 for instance). However, we agree that the way the objectives were phrased was ambiguous and have revised them at the end of the Background section to ensure better alignment with the rest of the manuscript (lines 107-113). They now read as follows:

"This article examines the representativeness of indirectly recruited participants in a nationwide cohort such as the Swiss Cohort of Healthcare Professionals and Informal Caregivers (SCOHPICA) [14,15] by comparing socio-demographic and socio-professional characteristics of its 2022-2023 baseline participants with those from other Swiss data sources. In doing so, it aims to assess whether indirect recruitment can constitute a practical and scientifically informative approach for studying healthcare professionals in the absence of comprehensive and up-to-date national registers, while evaluating the extent to which the resulting sample reflects the target population."

Using statistical tests for the comparative analysis also implies that we had to adapt the vocabulary in the Results section. This is for instance visible in lines 208-213, as well as in lines 221, 227 and 228. It also enabled us to be more precise in the comparison by spoken language, as shown in line 218. In sum, we are grateful to the reviewer for orienting us toward a path that was not initially planned, but that ultimately helped us to evaluate more formally how similar the SCOHPICA sample is compared to the external data sources across the different characteristi

---

VERSION 2 - REVIEW

Reviewer 1

Name Blake, Helen

Affiliation London School of Hygiene & Tropical Medicine

Date 11-May-2026

COI

Thank you for the opportunity to review this revised manuscript. The authors have done a lot of work to address the comments from all reviewers, and I appreciate the effort made, especially the work done to develop and present the new representativeness ratings.

The original manuscript used qualitative comparisons based on group consensus (i.e. 'no clear-cut difference', 'a trend towards a difference', or 'a meaningful difference'). The revised manuscript instead uses statistical tests and a 95% confidence level to dichotomise comparisons as 'significantly' different or not. My key concern with this new approach is regarding the issue of multiple testing, as the more statistical tests that are carried out, the higher the risk of false positives. At the very least, the issue of multiple testing needs to be addressed in the limitations.

Also, I worry that removing the qualitative comparisons from the manuscripts means losing consideration of whether differences are scientifically important. In the response to reviewers, the authors mention that their results don't change much after switching methodologies – including both as a form of triangulation could help identify whether any of the 'significant differences' may or may not be spurious.

Reviewer 3

Name Dawson, Jeremy

Affiliation The University of Sheffield, Sheffield University Management School

Date 11-May-2026

COI

Thank you for your attention to the previous round of reviews. I think you have addressed many of the concerns well and this is closer to being ready for publication. However, I have one further comment.

You addressed my concern about the hypothesis of representativeness not being tested, by adding in significance tests of characteristics of the sample with those of the population. While that is helpful, it doesn't quite answer the question in the most meaningful way due to the impact of sample size. To explain this, if you had a really large sample, it would very

likely produce significant differences, even if there were only very slight discrepancies from the population; on the other hand, a small sample size would never appear significantly different, even if there was quite a large difference. Therefore it is important to discuss the actual differences in terms of the effect size (i.e. actual difference from the population in a characteristic), not just the statistical significance. In my previous review I suggested anything more than a 10% difference might suggest a lack of representativeness. That figure is arbitrary, and I'm not suggesting you use it as a cut-off, but as a potential guide to indicate what the actual level of difference was. (You may choose a different figure, which is absolutely fine if you can justify it.)

Other than this, I am happy with the changes you have made.

VERSION 2 - AUTHOR RESPONSE

Reviewer: 3

Thank you for your attention to the previous round of reviews. I think you have addressed many of the concerns well and this is closer to being ready for publication. However, I have one further comment.

You addressed my concern about the hypothesis of representativeness not being tested, by adding in significance tests of characteristics of the sample with those of the population. While that is helpful, it doesn't quite answer the question in the most meaningful way due to the impact of sample size. To explain this, if you had a really large sample, it would very likely produce significant differences, even if there were only very slight discrepancies from the population; on the other hand, a small sample size would never appear significantly different, even if there was quite a large difference. Therefore it is important to discuss the actual differences in terms of the effect size (i.e. actual difference from the population in a characteristic), not just the statistical significance. In my previous review I suggested anything more than a 10% difference might suggest a lack of representativeness. That figure is arbitrary, and I'm not suggesting you use it as a cut-off, but as a potential guide to indicate what the actual level of difference was. (You may choose a different figure, which is absolutely fine if you can justify it.)

Other than this, I am happy with the changes you have made.

We thank the reviewer for this constructive comment and agree that statistical significance alone was insufficient to assess representativeness, given the influence of sample size on p-values.

To address this concern, we complemented the two-proportion z-tests with an assessment of absolute differences in proportions between SCOHPICA-HCP and the external data sources. Combining these quantitative and semi-quantitative approaches allowed us to identify discrepancies that were not only statistically significant but also

potentially meaningful in magnitude. We retained the reviewer's suggested threshold of 10 percentage points, as it appeared reasonable for this exploratory comparison and we did not identify a more justified alternative. However, we also explicitly acknowledge that this threshold should be interpreted as a pragmatic aid to interpretation rather than as a definitive criterion for representativeness.

We revised the manuscript accordingly by extending the Methods section (lines 179-184, reproduced below), updating the tables and table notes, expanding the Results section (lines 225-226, 229-231, 237-238, and 241-243), revising the opening paragraph of the Discussion (lines 275-283), and adding a limitation paragraph on statistical testing, multiple comparisons, and the pragmatic nature of the magnitude threshold (lines 370-377). This addition strengthens the manuscript by showing that some statistically significant discrepancies are more meaningful than others, thereby reducing the risk of overinterpreting small but statistically significant differences.

"To complement statistical testing and account for the influence of sample size on p-values, absolute differences in proportions were also examined. Differences exceeding 10 percentage points were considered indicative of potentially meaningful discrepancies in representativeness, while recognising that this threshold is intended as a pragmatic interpretive guide rather than a formal cut-off. Comparisons were therefore interpreted by jointly considering statistical significance and the magnitude of observed differences."

Reviewer: 1

Thank you for the opportunity to review this revised manuscript. The authors have done a lot of work to address the comments from all reviewers, and I appreciate the effort made, especially the work done to develop and present the new representativeness ratings.

The original manuscript used qualitative comparisons based on group consensus (i.e. 'no clear-cut difference', 'a trend towards a difference', or 'a meaningful difference'). The revised manuscript instead uses statistical tests and a 95% confidence level to dichotomise comparisons as 'significantly' different or not. My key concern with this new approach is regarding the issue of multiple testing, as the more statistical tests that are carried out, the higher the risk of false positives. At the very least, the issue of multiple testing needs to be addressed in the limitations.

Also, I worry that removing the qualitative comparisons from the manuscripts means losing consideration of whether differences are scientifically important. In the response to reviewers, the authors mention that their results don't change much after switching methodologies – including both as a form of triangulation could help identify whether any of the 'significant differences' may or may not be spurious.

We thank the reviewer for this constructive comment and agree that relying only on statistical significance may lead to overinterpretation, particularly in the context of multiple comparisons.

To address this, we did not revert fully to the previous qualitative consensus-based comparisons, but introduced a complementary semi-quantitative assessment based on absolute differences in proportions, following the suggestion made by the other reviewer. The revised approach therefore combines statistical testing with an assessment of whether observed differences are meaningful in magnitude.

We revised the manuscript accordingly by extending the Methods section (lines 179-184), updating the tables and table notes, expanding the Results section (lines 224-226, 229-231, 237-238, and 241-243), revising the opening paragraph of the Discussion (lines 275-283), and adding a limitation paragraph explicitly addressing the limitations of statistical testing, including sample size, multiple comparisons, and the pragmatic nature of the magnitude threshold (lines 370-377, reproduced below).

We believe this combined approach addresses the reviewer's concern by helping distinguish statistically significant differences that are limited in magnitude from those more likely to reflect scientifically meaningful discrepancies, thereby reducing the risk of overinterpreting potentially spurious findings.

"A further limitation concerns the comparison methods themselves. Statistical testing helped identify differences between SCOHPICA-HCP and external data sources, but p-values are influenced by sample size and the large number of comparisons performed increases the risk of false-positive findings. Conversely, the predefined magnitude threshold was pragmatic and necessarily somewhat arbitrary, and should be interpreted as an aid to interpretation rather than as a definitive criterion for representativeness. Combining statistical testing with an assessment of absolute differences therefore provided a more nuanced interpretation of discrepancies between SCOHPICA-HCP and external data sources."

VERSION 3 - REVIEW

**Reviewer**	**1**
**Name**	**Blake, Helen**
**Affiliation**	**London School of Hygiene & Tropical Medicine**
**Date**	**04-Jun-2026**
**COI**	

Thank you for the opportunity to review manuscript after the second round of revisions. The manuscript has been strengthened by additionally considering the absolute differences in proportions. Thank you for addressing my concerns regarding multiple hypothesis testing. I have no further comments.

Reviewer 3

Name Dawson, Jeremy

Affiliation The University of Sheffield, Sheffield University Management School

Date 04-Jun-2026

COI

Thank you for your attention to my previous comments. I believe you have addressed these well now.
